# Complete Genome Sequence of *Mycoplasma hyorhinis* Strain SK76

**DOI:** 10.1128/genomeA.00101-12

**Published:** 2013-02-07

**Authors:** Steve Goodison, Virginia Urquidi, Dibyendu Kumar, Leticia Reyes, Charles J. Rosser

**Affiliations:** a Cancer Research Institute, MD Anderson Cancer Center Orlando, Orlando, Florida, USA; b Waksman Institute of Microbiology, Rutgers, the State University of New Jersey, Piscataway, New Jersey, USA; c College of Dentistry, University of Florida, Gainesville, Florida, USA; d Section of Urologic Oncology, MD Anderson Cancer Center Orlando, Orlando, Florida, USA

## Abstract

*Mycoplasma hyorhinis* is a eubacterium belonging to the *Mollicutes* class and is responsible for porcine respiratory and arthritic diseases. It is also the major contaminant of mammalian tissue cultures in laboratories worldwide. Here, we report the complete genome sequence of *M. hyorhinis* strain SK76.

## GENOME ANNOUNCEMENT

*Mycoplasma hyorhinis* is a swine pathogen that causes chronic disease after its initial colonization of mucosal surfaces of the respiratory tract (1). *M. hyorhinis* can also infect human cells under laboratory conditions (2–4), and chronic infection has been shown to cause irreversible changes that are associated with malignant transformation (5, 6). Here, we report the complete genome sequence of *M. hyorhinis* strain SK76 (clonal isolate 8II), an arthritogenic strain that was derived from a naturally infected disease site (7–9). While this study was in progress, the genome sequences of three other *M. hyorhinis* strains were reported (10–12).

The genomic libraries were prepared (2- to 4-kb inserts) using 454 Life Sciences (Roche Diagnostics) standard protocols, and their sequences were determined by sequencing on the 454 GS-FLX Titanium platform at the Interdisciplinary Center for Biotechnology Research, University of Florida, Gainesville, FL. The resulting sequence reads (52-fold coverage of the genome) were assembled into a scaffold using the 454 Newbler software and they were ordered using optical mapping (13) (OpGen, Inc., Madison, WI). The finishing sequences from duplicate PCR products were derived from a fosmid library (pEpiFOS-5 vector/EPI100 host cells) using Sanger sequencing, and they were mapped to the scaffold using the Consed software package (CodonCode Corporation, Centerville, MA). The genome sequence was annotated using the Rapid Annotations using Subsystems Technology (RAST) Prokaryotic Genome Annotation Server (http://www.nmpdr.org/FIG/wiki/view.cgi/Main/RAST).

The genome of *M. hyorhinis* strain SK76 is composed of a single circular chromosome of 836,897 bp with an overall G+C content of 26%. The genome contains 753 putative coding DNA sequences (CDSs). The average size of a CDS in this genome is 1,002 bp, with an average of one gene every 1,110 bp. This is similar to the gene sizes and densities seen with the smaller *M. genitalium* genome, as well as with the genomes of many species of Gram-positive bacteria, which are far larger than *M. hyorhinis*. The genome contains one copy of each of the 5S, 16S, and 23S rRNA genes, which are not organized in an operon. The 16S and 23S rRNA genes are adjacent, but the 5S rRNA gene is located in a distant region.

Several species of mycoplasma contain a genetic system that allows them to undergo high-frequency surface antigenic variations. The variable surface lipoprotein (*vlp*) locus contains up to seven distinct single-copy *vlp* genes (7). The size variation of Vlp products results from the insertion or deletion of tandemly repeated intragenic sequences that expand or contract the surface Vlp C-terminal region (14). The *vlp* locus of *M. hyorhinis* SK76 clonal isolate 8II contains all seven *vlp* genes, arranged in the order 5′-*vlpD-vlpE-vlpF*-insertion sequence (IS)*-vlpG-vlpA-*IS*-vlpB-vlpC*-3′. *M. hyorhinis* strain MCLD contains only 4 *vlp* genes (*vlpB* to *vlpE*), and *M. hyorhinis* strain GDL contains six *vlp* genes (*vlpA* to *vlpF*). *M. hyorhinis* hub-1 also contains all seven genes in the *vlp* locus.

### Nucleotide sequence accession number.

The complete genome sequence of *M. hyorhinis* strain SK76 has been deposited in the NCBI GenBank database under the accession no. CP003914.
